# A retrospective analysis of biochemical and haematological parameters in patients with eating disorders

**DOI:** 10.1186/s40337-017-0158-y

**Published:** 2017-10-02

**Authors:** Leanne J. Barron, Robert F. Barron, Jeremy C. S. Johnson, Ingrid Wagner, Cameron J. B. Ward, Shannon R. B. Ward, Faye M. Barron, Warren K. Ward

**Affiliations:** 1 Brisbane City Doctors Medical Practice, Brisbane, QLD Australia; 2 Eating Disorders Multidisciplinary Clinic, Queensland University of Technology, Brisbane, QLD Australia; 3 Riverina-Murray Institute of Higher Education, Wagga Wagga, NSW Australia; 4 Royal Brisbane and Women’s Hospital, Brisbane, QLD Australia; 5 School of Public Health and Social Work, Queensland University of Technology, Brisbane, QLD Australia; 6 Lady Cilento Children’s Hospital, Brisbane, QLD Australia; 7 University of Queensland, Brisbane, QLD Australia; 8 Mount Erin College, Wagga Wagga, NSW Australia; 9 Eating Disorders Service, Royal Brisbane and Women’s Hospital, Brisbane, QLD Australia; 10 School of Medicine, University of Queensland, Brisbane, QLD Australia; 11 Mater Medical Research Institute, Brisbane, Australia; 12 Queensland Paediatric Cardiac Research Group, Queensland, Australia

**Keywords:** Retrospective, Eating disorders, Biochemical, Haematological, Parameter, Reference interval, General practice, Primary care

## Abstract

**Background:**

The objective of the study was to determine whether levels of biochemical and haematological parameters in patients with eating disorders (EDs) varied from the general population. Whilst dietary restrictions can lead to nutritional deficiencies, specific abnormalities may be relevant to the diagnosis, pathogenesis and treatment of EDs.

**Methods:**

With ethics approval and informed consent, a retrospective chart audit was conducted of 113 patients with EDs at a general practice in Brisbane, Australia. This was analysed first as a total group (TG) and then in 4 ED subgroups: Anorexia nervosa (AN), Bulimia nervosa (BN), ED Not Otherwise Specified (EDNOS), and AN/BN. Eighteen parameters were assessed at or near first presentation: cholesterol, folate, vitamin B12, magnesium, manganese, zinc, calcium, potassium, urate, sodium, albumin, phosphate, ferritin, vitamin D, white cell count, neutrophils, red cell count and platelets. Results were analysed using IBM SPSS 21 and Microsoft Excel 2013 by two-tailed, one-sample t-tests (TG and 4 subgroups) and chi-square tests (TG only) and compared to the population mean standards. Results for the TG and each subgroup individually were then compared with the known reference interval (RI).

**Results:**

For the total sample, t-tests showed significant differences for all parameters (*p* < 0.05) except cholesterol. Most parameters gave results below population levels, but folate, phosphate, albumin, calcium and vitamin B12 were above. More patients than expected were below the RI for most parameters in the TG and subgroups.

**C**onclusions**:**

At diagnosis, in patients with EDs, there are often significant differences in multiple haematological and biochemical parameters. Early identification of these abnormalities may provide additional avenues of ED treatment through supplementation and dietary guidance, and may be used to reinforce negative impacts on health caused by the ED to the patient, their family and their treatment team (general practitioner, dietitian and mental health professionals). Study data would support routine measurement of a full blood count and electrolytes, phosphate, magnesium, liver function tests, ferritin, vitamin B12, red cell folate, vitamin D, manganese and zinc for all patients at first presentation with an ED.

## Plain English summary

There is a need for developing basic knowledge at the primary care level of abnormalities in blood tests found in patients suffering from eating disorders (EDs). This retrospective analysis compared the levels of 18 biochemical and haematological parameters in patients with EDs to those for the general population. For the parameters studied, statistical analysis showed significant variations (*p* ≤ 0.05) from the general population for all but one of the parameters (cholesterol). Analysis using reference intervals showed significantly more cases than expected outside the normal range for 12 parameters. This information could be useful in the ongoing treatment of EDs and provide guidance for nutritional programs.

## Background

There is limited information in the literature regarding abnormalities in measured biochemical and haematological parameters in patients with eating disorders (EDs) in the primary care setting [1]. This retrospective study was undertaken to investigate whether levels of selected biochemical and haematological parameters in patients diagnosed with EDs were significantly different from the general population [2]. The purpose was not to minimise the importance of generally accepted causes or treatments of EDs but rather to identify those pathological abnormalities that would support the diagnosis and aid in the management of an ED. Cases of clinical anorexia nervosa (AN) are not common in primary care practices. The incidence of severe EDs in the United Kingdom has been estimated as 1–2 per practice although less serious cases may reach levels of 3% to 5% of the population [3]. The most rapid increases in prevalence are now occurring in children under the age of 12 [4, 5]. Study data were obtained from patient files of a single general practitioner (GP) who acts as a primary care “referral base” for patients with EDs. This study not only represents one of the larger study groups of patients with EDs but is particularly unique in that it is from the ambulatory primary care setting as opposed to most published data which are derived from secondary or tertiary care facilities [3].

EDs are complex multifactorial conditions [4]. Traditionally accepted causes of EDs include psychological issues (eg body image and peer pressure) and environmental issues (eg family background) [6]. Genetic factors are increasingly being investigated and may explain the familial nature of EDs [7–10]. While dietary restriction is the only proven causative factor in ED patients, only a small percentage of those on diets develop an ED [4, 11]. The trigger stimulating EDs in susceptible patients is unknown, although it is possible that biochemical abnormalities including nutritional parameters may not just be the result of the ED, but may also play a role in the initiation of the ED. [12, 13] Medical stabilization and nutritional rehabilitation are important determinants of short-term outcomes for patients with EDs [4, 5, 14–16]. A literature review [1] suggests the need for basic nutritional knowledge about AN with the aim to provide a personalised, evidence based treatment plan.

A number of studies support the measurement of biochemical and haematological parameters as a guide to diagnosis and treatment of EDs. Rukgauer et al. suggested measurement of trace elements such as copper, manganese, selenium and zinc be undertaken to guide diagnosis and therapy [17]. The Society for Adolescent Medicine stated that significant deficiencies of calcium, vitamin D, folate, vitamin B12, and other minerals were found in those suffering from EDs [16]. Miller et al. indicated that for college-aged women with AN, levels of sodium, potassium and calcium were low, and serum albumin levels were elevated [18]. Amongst patients with EDs measurement of magnesium, complete blood count, serum electrolytes, calcium, creatinine, blood urea nitrogen, phosphate, ferritin, albumin, vitamin B12, folate, erythrocyte sedimentation rate (ESR), liver function tests, random blood glucose, zinc and manganese levels and routine urinalysis have all been advocated [4, 19–22]. Routine supplements of thiamine, phosphate, potassium,zinc and other minerals have been recommended for treatment of AN [11, 15, 16, 21].

## Methods

Ethics approval from the Royal Australian College of General Practitioners, consent from the Principal of the medical practice and patient informed consent were obtained prior to data collection. Data were collected and de-identified prior to analysis.

### Data collection and analysis

Retrospective data were obtained from the first consultation with the GP or as soon as possible after that time. Most of the patients presented with a known diagnosis of an ED but no data were available as to the time interval between the onset of symptoms of the ED and the first consultation with the GP. As a prerequisite of ethics approval, no data could be collected that would permit the GP to identify the patient. To comply with this ethics requirement, no clinical data other than diagnosis were collected.

Blood levels of cholesterol, red cell folate, vitamin B12, magnesium, manganese, zinc, calcium, potassium, urate, sodium, albumin, phosphate, ferritin, vitamin D, white blood cells, neutrophils, red blood cells and platelets were collected. Body Mass Index (BMI) was calculated from height and weight, taken wearing light clothing and using standardized equipment. BMI data were included to give some additional information regarding patients’ clinical status, but no data were available regarding their treatment status, duration of illness, comorbidities, medications or supplement use.

Data were accessed by members of the study team other than the GP, de-identified, and analysed using numerical reference ID numbers only. Total group (TG) size was 113, but was slightly lower for some parameters due to availability of data. Patient test results were collated for each parameter, then means, standard deviations, reference intervals (RIs), t-test scores, chi-square (TG only) and probability levels determined. A minimum significance level of ≤0.05 was accepted throughout the study.

Data were collated and analysed for the TG, then subdivided into 4 ED subgroups: Anorexia Nervosa (AN); Bulimia Nervosa (BN); EDs not otherwise specified (EDNOS); and patients with a history of AN who later presented with BN (AN/BN), as opposed to those with primary BN [23]. Diagnoses were based on the criteria in DSM-IV [24] by the GP or a treating psychiatrist or psychologist. Table 1 provides the ages and relative subgroup distribution (60.2% AN; 15.0% BN; 10.6% EDNOS; and 14.2% AN/BN). Nineteen of the 24 patients aged under 18 years were diagnosed as AN, representing 28% of the AN subgroup and 17% of the TG. Seven patients were under 15 years of age.

**Table 1:** Age distribution of total group and subgroup patients at first presentation

Group	n	Mean age (SD) (years)	Age range (Years)	*n* < 15 years	n 15–17 years	n 18–20 years	n 21–30 years	*n* > 30 years
Anorexia	68	20.86 [± 5.69]	10.7–40.2	6	13	22	21	6
Bulimia	17	25.44 [± 8.69]	13.4–48.1	1	2	2	8	4
Ednos	12	24.99 [±4.67]	16.3–32.4	0	2	1	8	1
AN/BN	16	26.86 [±7.93]	18.5–45.0	0	0	4	8	4
Total group	113	22.84 {±6.94]	10.7–48.1	7	17	29	45	15

SD = standard deviation *n* = number of patients

Data were analysed using IBM SPSS 21 and Microsoft Excel 2013 to calculate TG and subgroup means and SDs for each clinical parameter which were then compared to those for the general population. The two-tailed, one-sample t-test was used to determine whether results were statistically significant. Results were then further analysed using RI data, with the numbers of patients above or below the RI being compared to statistically expected numbers. Results for the TG were further analysed using the goodness of fit chi square test. Only raw data were used to analyse subgroups RI results because of their small numerical size. Chi squared results when used were adjusted using the Yates correction.

#### Reference intervals

For any measured pathological parameter, RIs are defined as two standard deviations above and below the mean within a given population [25]. The setting of these intervals is achieved by standardization on a large and appropriate population group, overseen in Australia by the National Association of Testing Authorities (NATA) [26]. The Australian Association of Clinical Biochemists (AACB) has a program to establish harmonised RIs for Australia and New Zealand [27]. Consequently, only minor variations in RIs are seen between pathology providers [28]. The RI data utilised were provided by the pathology company [29] which undertook the majority of the routine testing of the patients involved.

Unlike other RIs, the cholesterol RI has been weighted to “desirable” ranges as opposed to a population RI. For folate and vitamin B12, upper levels of the RI were not available from the pathology company, which provided only minimums [30]. The red cell folate minimum for the pathology provider changed from >900 nmol/L to >150 nmol/L on 21 January 2013 when the test was changed [30]; test results obtained after that date were not included in the study resulting in a smaller sample size of 78 for this parameter only. The Australian Aborigine and Torres Strait Islanders Health Survey 2012–13, [31] used an RI of 776 to 1784 nmol/L for red cell folate, which was adopted in this study. Vitamin B12 test material provided by the pathology company gave an RI of 138 to 652 pmol/L [32].

## Results

**Fig. 1:**
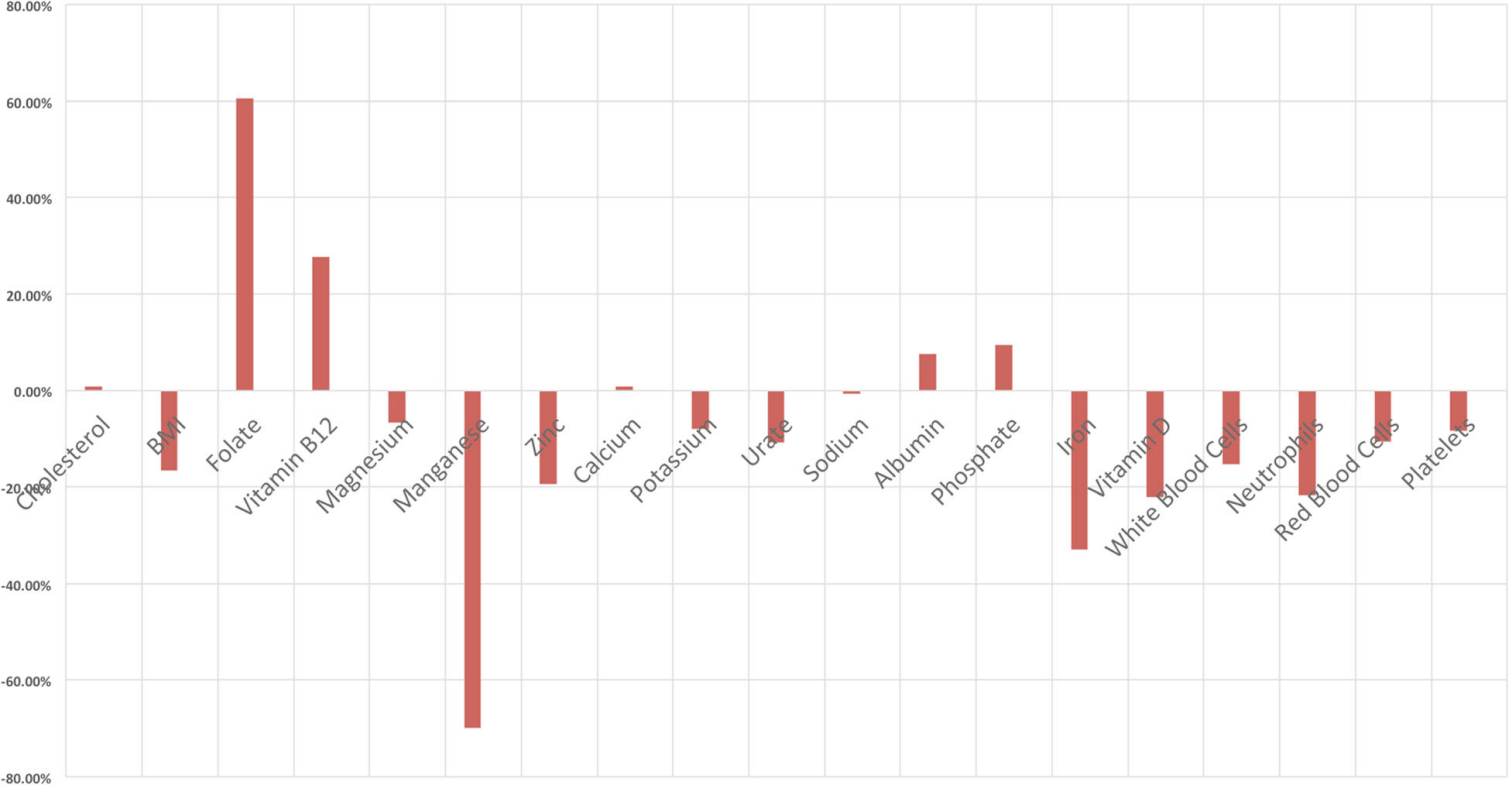
Percentage differences between total group and population mean scores, all parameters

Means, SDs and t-test significance levels for all TG parameters are given in Table 2 and Fig. 1, population means being taken as the midpoint of the RIs. Probability levels for means and SDs for the TG were significant at <0.05 for all parameters except cholesterol.

**Table 2:** Comparison of clinical indices between primary care eating disorder patients and general population

Parameter	Unit of measure	Reference interval	Population mean (SD)	Study mean (SD)	Study result range	t score	*p*-value
Cholesterol	mmol/L	3.9–5.5	4.7 (±0.4)	4.74 (±1.03)	2.7 to 8.5	0.41	0.68
Body mass index	Kg/m^2^	18.5–24.9	21.75(±1.63)	18.14 (±3.11)	12.7 to 30.4	−11.55	**≤0.001**
Red cell folate^a^	nmol/L	776–1784	1280 (±252)	2055 (±584)	627 to 4103	11.73	**≤0.001**
Vitamin B12	pmol/L	138–652	393 (±126)	502 (±278)	142 to 1480	3.77	**≤0.001**
Magnesium	mmol/L	0.7–1.1	0.9 (±0.1)	0.84 (±0.08)	0.7 to 1.1	−7.87	**≤0.001**
Manganese	nmol/L	10.0–45.0	27.5 (±8.75)	8.3 (±3.07)	4.0 to 21.0	−65.39	**≤0.001**
Zinc	umol/L	9.0–19.0	14.0 (±2.5)	11.31 (±1.88)	6.7 to 18.5	−17.46	**≤0.001**
Calcium	mmol/L	2.1–2.55	2.33 (±0.11)	2.31 (±0.10)	2.11 to 2.51	2.08	**≤0.05**
Potassium	mmol/L	3.5–5.5	4.5 (±0.50)	4.14 (±0.50)	2.5 to 5.8	−7.48	**≤0.001**
Urate	mmol/L	0.15–0.4	0.28 (±0.06)	0.25 (±0.08)	0.13 to 0.48	−3.9	**≤0.001**
Sodium	mmol/L	135–145	140 (±2.5)	139.13 (±2.91)	130 to 145	−3.1	**≤0.01**
Albumin	g/L	37.0–48.0	42.5 (±2.75)	45.72 (±3.4)	41.0 to 53.0	9.8	**≤0.001**
Phosphate	mmol/L	0.8–1.5	1.15(±0.175)	1.26 (±0.18)	0.9 to 1.9	6.47	**≤0.001**
Ferritin	μg/L	30–150	90 (±21.25)	61.22 (±58.7)	4.0 to 380.0	−5.14	**≤0.001**
Vitamin D	nmol/L	50–150	100 (±25.0)	78 (±26.23)	21.0 to 189.0	−8.55	**≤0.001**
White blood cells	10^9^/L	3.5–11.0	7.25 (±1.88)	6.14 (±2.1)	2.3 to 16.6	−5.59	**≤0.001**
Neutrophils	10^9^/L	1.5–7.5	4.5 (±1.5)	3.53 (±1.43)	1.1 to.8.15	−7.18	**≤0.001**
Red blood cells	10^12^/L	3.9–5.6	4.75 (±0.43)	4.25 (±0.38)	3.0 to 4.9	−13.93	**≤0.001**
Platelets	10^9^/L	150–400	275 (±62.5)	252 (±66.0)	108 to 530	−3.69	**≤0.001**

^a^As indicated in the text, only patients tested on this parameter before a test change was made by the pathology company on 21/01/13 were included in the study

SD = standard deviation

Results for subgroups are given in Table 3. AN subgroup results indicated all parameters except cholesterol, calcium, and sodium were significantly different from levels in the general population. For BN, parameters not significant were cholesterol, vitamin B12, urate, sodium, phosphate, white blood cells, and neutrophils; for the EDNOS subgroup, cholesterol, vitamin B12, calcium, sodium, and platelets were not significant, and for the AN/BN subgroup, cholesterol, vitamin B12, calcium, urate, albumin, and platelets were not found to be significantly different from the population levels.

**Table 3:** Analysis of measured clinical parameters for subgroups

	Anorexia subgroup		Bulimia subgroup		Ednos subgroup		AN/BN subgroup	
**Parameter**	**Mean (SD)**	***p*** **value**	**Mean (SD)**	***p*** **Value**	**Mean (SD)**	***p*** **value**	**Mean (SD)**	***p*** **value**
Cholesterol	4.72(±1.11)	0.88	4.59(±0.69)	0.52	4.70(±0.9)	0	5.03(±1.02)	0.22
Bmi	16.45(±1.63)	**≤0.001**	20.52(±1.95)	**0.029**	22.15(±3.64)	0.73	20.19(±3.21)	0.11
Folate	2100(±700)	**≤0.001**	1903(±373)	**≤0.001**	2102(±404)	≤**0.001**	2025(±415)	**≤0.001**
Vit B12	527(±271)	**≤0.01**	440(±240)	0.43	393(±172)	0.97	571(±371)	0.07
Magnesium	0.84(±0.08)	**≤0.001**	0.81(±0.07)	**≤0.001**	0.85(±0.06)	**0.02**	0.81(±0.06)	**≤0.001**
Manganese	7.79(±2.42)	**≤0.001**	7.19(±2.07)	**≤0.001**	8.17(±2.73	**0.001**	11.4(±4.24)	**≤0.001**
Zinc	11.0(±1.76)	**≤0.001**	11.95(±2.07)	**0.003**	11.85(±1.39)	**0.001**	11.45(±1.94)	**≤0.001**
Calcium	2.32(±0.11)	0.42	2.27(±0.09)	**0.014**	2.31(±0.06)	0.29	2.32(±0.12)	0.753
Potassium	4.1(±0.53)	**≤0.001**	4.24(±0.46)	**0.033**	4.25(±0.25)	**0.007**	4.1(±0.53)	**0.01**
Urate	0.24(±0.07)	**≤0.001**	0.28(±0.08)	0	0.24(±0.06)	**0.049**	0.26(±0.09)	0.403
Sodium	139.2(±3.26)	0.052	139.35(±2.54)	0.15	139.09(±2.02)	0.16	138.6(±2.03)	**0.017**
Albumin	46.25(±2.92)	**≤0.001**	45.47(±3.87)	**0.006**	44.82(±2.69)	**0.016**	44.33(±4.47)	0.133
Phosphate	1.27(±0.19)	**≤0.001**	1.19(±0.12)	0.19	1.24(±0.14)	**0.048**	1.24(±0.16)	**0.04**
Ferritin	68.2(±61.0)	**0.02**	46(±33)	**0.02**	54.0(±28.0)	**≤0.001**	48.0(±72.0)	**0.034**
Vitamin D	79.7(±21.95)	**≤0.001**	72.3(±26.2)	**≤0.001**	79.7(±25.7)	**0.02**	75.2(±38.1)	**0.02**
W.B.C.	6.13(±2.32)	**≤0.001**	6.68(±1.83)	0.22	6.27(±1.68)	**0.04**	5.53(±1.35)	**≤0.001**
Neutrophils	3.51(±1.46)	**≤0.001**	3.86(±1.5)	0.09	3.5(±1.26)	**0.01**	3.05(±0.87)	**≤0.001**
R.B.C.	4.25(±0.39)	**≤0.001**	4.2(±0.33)	**≤0.001**	4.19(±0.43	**0.019**	4.34(±0.39)	**≤0.001**
Platelets	256(±71)	**0.032**	236(±45.7)	**0.003**	244(±56.7)	0.08	258(±62.5)	0.29

Significant *p*-values shown in BOLD type SD = Standard deviation p = probability value BMI = body mass index

W.B.C = white blood cells R.B.C. = red blood cells EDNOS = eating disorders not otherwise stated

AN/BN = initial anorexia eating disorder diagnosis, then bulimia at a later stage

**Table 4:** Probability values, all results, for total group patients outside reference intervals

Parameter	Number of patients (n)	Expected frequency outside RIs	Observed frequency outside RIs	CHI SQUARE	*p*-Value
Cholesterol	113	5.65	46	281.07	**≤0.001**
Body mass index	102	5.1	58	557.88	**≤0.001**
Folate	78^a^	3.9	54	630.81	**≤0.001**
Vitamin B12	96	4.8	16	23.85	**≤0.001**
Magnesium	110	5.5	0	6.55	**0.013**
MANGANESE	106	5.3	76	929.82	**≤0.001**
Zinc	108	5.4	5	0.14	0.71
Calcium	108	5.4	3	1.56	0.21
Potassium	108	5.4	11	4.82	**0.028**
Urate	108	5.4	6	0.01	0.92
Sodium	108	5.4	8	0.82	0.36
Albumin	108	5.4	24	60.67	**≤0.001**
Phosphate	112	5.6	6	0.01	0.92
Ferritin	104	5.2	44	282.1	**≤0.001**
Vitamin D	104	5.2	16	20.4	**≤0.001**
White blood cells	112	5.6	13	8.50	**0.003**
Neutrophils	112	5.6	8	0.69	0.41
Red blood cells	112	5.6	16	17.17	**≤0.001**
Platelets	112	5.6	6	0.01	0.92

RIs = reference intervals Significant *p*-values shown in **BOLD** type

Observed frequency = number in study outside RIs; >2 SD’s outside the mean for that parameter

^a^As indicated in the text, only patients tested on this parameter before a test change was made by the pathology company on 21/01/13 were included in the study

**Fig. 2:**
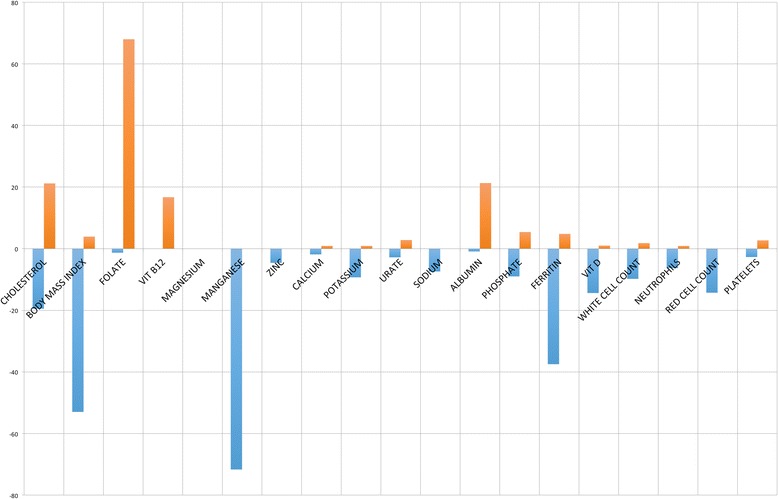
% Total group results above and below population reference intervals

**Fig. 3:**
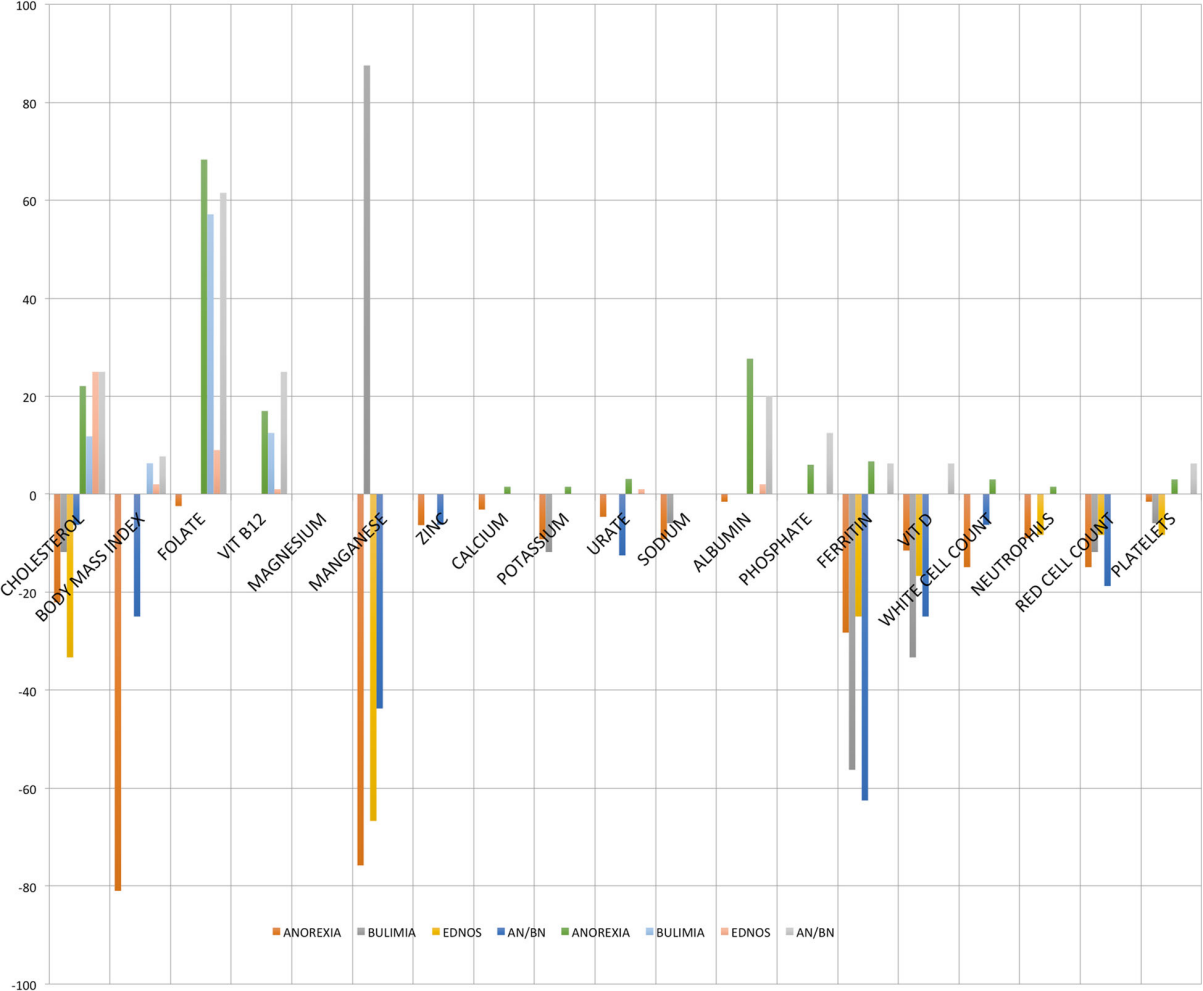
% subgroup results above and below population reference intervals

Results were then analysed to determine the frequency of patient results outside the RI (Table 4). BMI is included for clinical interpretation. For the TG, manganese showed the largest number of results below the RI, followed by ferritin, cholesterol, vitamin D, red blood cells, potassium, sodium, white cell count, neutrophils and red cell count. More than 60% of patients in the TG had levels of red cell folate above the RI, over 20% of patients had cholesterol and/or albumin levels above the RI and 20% of patients had cholesterol levels below the RI. Figure 2 shows percentages for the TG above and below the RI for each parameter. The percentage of cases above or below the RI for each subgroup is shown in Fig. 3.

## Discussion

This study demonstrated statistically significant t test variations for the TG in comparison to the population data in all 18 parameters studied except cholesterol. Although the t test result for cholesterol was not significant, 40% were outside the RI, with relatively equal results above and below the RI. T test results for the subgroups also showed significant variations.

AN is a condition characterized by protein energy undernutrition, and sufferers may show deficiencies in minerals and electrolytes largely derived from protein sources such as zinc, potassium, phosphate and calcium, in addition to vitamin D deficiency [33].

Particularly striking was the consistently low level of manganese, considered a factor in mental health as early as the 1920s [34]. Manganese is important for enzymes involved in carbohydrate metabolism, and for connective tissue/cell membrane integrity. Manganese deficiency may result in osteoporosis, impaired insulin production, alteration in lipoprotein metabolism, an impaired oxidant defence system, fatigue, and abnormalities in growth factor metabolism [35]. It can also be associated with epilepsy, dizziness and schizophrenia [36–39]. However, neurological toxicity has been reported at high doses, including Parkinson’s disease-like side effects [40] therefore any supplementation requires careful monitoring. A World Health Organization (WHO) report [41] suggested that only very rarely has research found significant deficiency in the level of manganese in humans - which suggests the low manganese in our population may be very significant.

Zinc levels were significantly reduced, which has previously been reported in patients with AN and those suffering from schizophrenia [11]. Birmingham et al. found that AN patients treated with zinc supplements showed a rate of BMI gain twice that of a placebo group [42]. Zinc is critical for the functioning of enzyme reactions including neurotransmitter synthesis [43]. Low levels of zinc can result in altered taste perceptions, loss of smell and a decrease in appetite [11]. A 2015 nursing home study demonstrated improved cognitive performance and mood in patients with higher levels of serum zinc [44]. It has been suggested that all patients with AN should be given zinc supplements [21].

Phosphate is important for cellular energy (ATP formation) and its deficiency is of particular concern during re-feeding. Although serum phosphate may be initially normal, body stores are likely to be low and increased metabolism with re-feeding can cause precipitous falls, with consequent muscle weakness and risk of cardiac failure [33]. Excessive intake of phosphate can lead to bone impairment and ageing [45]. The higher than expected phosphate levels in this study may relate to variability in severity and/or phosphate supplementation.

Magnesium is a co-factor in over 300 enzymatic reactions known to be important in the normal functioning of muscles, nerves, heart, bones, and the immune system, in regulating potassium fluxes and in the metabolism of calcium [46, 47]. Magnesium deficiency has also been linked to stress, anxiety [11], and excitability or depression [48]. Significant falls during re-feeding in EDs are well documented [49]). Therefore, although this study did not show any patients outside the RI the authors strongly recommend magnesium be routinely measured throughout diagnosis and treatment. Serum magnesium reflects less than 1% of body stores, however low serum levels are a simple and accurate means for assessment of acute changes in magnesium status [46].

Ferritin stores and transfers iron in a non-toxic form in the body. Iron is necessary for the production of haemoglobin and red blood cells. It is also considered critical for proper brain function and, as levels drop, depression and fatigue may develop [11]. Study results indicated that one third of TG patients scored below the population RI, while only a small percentage were high. As ferritin is an acute phase reactant however levels may not necessarily reflect iron stores [50, 51].

Although cholesterol levels failed to reach significance levels in the t-test analysis, it was noted that the TG showed 19% of patients to have cholesterol levels below the RI, and 21% above the RI. Theories have been proposed for the paradoxical elevation of cholesterol in those with severe EDs including being related to low levels of thyroid hormones resulting in reduced metabolism of existing cholesterol [52, 53]. Low levels of cholesterol have been associated with depression, anxiety and suicidal tendencies [54–58]. Impaired cholesterol synthesis may explain the low cholesterol in some individuals [58]. In vivo cholesterol synthesis is a complex process requiring multiple nutrients including manganese [39, 59]]. This is of particular significance in the context of the low manganese levels in the study population.

Reduced metabolism may explain the significantly elevated results for vitamin B12 and folate and the absence of low levels relative to the RIs. Both folate and vitamin B12 were found to be low prior to the introduction of voluntary folate additives in 1998, made compulsory by the Australian Government in 2009 [60]. The National Centre for Environmental Health in the US reported a small increase in serum Vitamin B12 following fortification [61]. It is accepted that high levels of folate and vitamin B12 are not normally a problem as long as they occur together, while low levels of folate are today rare and often not clinically monitored [62]. It has been shown that cells require vitamin B12 to utilize folic acid. Thus, if B12 is too low, folic acid may accumulate [63]. Vitamin B12 is essential for the formation of red blood cells and the health of nerve tissue [11].

Vitamin D levels were significantly reduced with implications for bone health, mental health and a potential susceptibility to the development of breast, lung and bowel cancers, multiple sclerosis, diabetes, auto-immune disorders, allergies and depression [64, 65]. Modan-Moses et al. reported a high prevalence of vitamin D deficiency in adolescents with EDs and suggested supplementation as required [66].

Calcium is important for nerve conduction, muscle contraction and metabolism of bones and teeth.(39). The study showed slightly elevated serum levels, but as ionised (free) calcium is considered a more reliable measure (49) this result is of uncertain significance.

Potassium and sodium were found to be significantly outside the RIs in the TG and AN subgroup while no significant deviation from the RI was found in other subgroups. Sodium and potassium are essential for cellular homeostasis, including proper functioning of nerves and muscles, including the heart. Hypokalaemia has been associated with cardiac arrhythmias which are believed to be a cause of mortality in EDs [67, 68].

Albumin is important in maintaining intravascular volume and transfer and metabolism of a large variety of molecules. The study found significantly higher levels in the TG, AN and AN/BN subgroups. Hyperalbuminaemia is commonly related to dehydration [69], although this explanation would seem somewhat incongruous in the ED population. Unfortunately, hydration levels of patients at the time of testing are unknown. Most patients with anorexia nervosa have normal serum albumin levels (49). The Minnesota starvation experiment demonstrated that even with a 23% reduction in body weight and muscle mass, serum albumin decreased only moderately. It has been noted that malnutrition in the absence of inflammation does not usually result in significant falls in serum albumin, probably because of compensatory reductions in albumin fractional catabolic rate and resting energy expenditure [70].

Urate, or uric acid, is the end product of purine metabolism in humans. It represents over 50% of the blood’s anti-oxidant capacity, but chronically high levels may be associated with gout and metabolic syndrome. It is not known whether elevated levels represent a risk factor or a protective factor. Production of lactate and ketone bodies can inhibit uric acid excretion, which may contribute to elevated levels in eating disorders [71].

Although haematological parameters in this study showed small variations from normal, they were statistically significant. Considering the risk of anaemia, neutropenia and thrombocytopenia in EDs, the value of monitoring the full blood count (FBC) is beyond question [22]. As with other results such as potassium, albumin, magnesium and phosphate, the low number of severe abnormalities may reflect a less severe degree of illness in patients presenting in a general practice as opposed to a hospital setting,

### Limitations of study

As this study is retrospective not all data were available for analysis. For some patients the initial consultation represented the first medical contact, whilst others had been within the medical system for some time. As such, previously initiated treatment regimes including dietary manipulations, additional supplements, psychiatric and medical interventions, all of which may be relevant, were not available. Full clinical information, including but not limited to vomiting, laxative intake, dietary intake, amount of exercise and intake of drugs and alcohol, were not included. Ethics approval was granted on the specific condition that patients who chose to participate would not be identifiable by the GP, and collection of this material could have threatened this condition.

Blood samples were not specifically collected in a fasting state (as this may jeopardise patient safety in an outpatient population) and there is no information available as to degrees of supplementation or oral intake prior to collection of any samples.

Although this medical practice has a disproportionately large number of patients diagnosed with EDs, sample sizes, particularly of some subgroups, were small. For this reason, statistical analysis was limited.

## Conclusions

The results suggest numerous biochemical and haematological abnormalities in patients with EDs. The benefits of establishing a program of nutritional rehabilitation for all sufferers of EDs are clear. Identification of specific deficiencies for each patient would permit personalised dietary guidance and supplementation, which could play a significant part in the treatment process. In addition to manganese the study strongly supports the collection of a routine full blood count, electrolytes, phosphate, liver function tests, cholesterol, vitamin B12, red cell folate, vitamin D, magnesium and zinc at initial presentation.

Recognition of these abnormalities supports the diagnosis of EDs and provides the opportunity to tailor treatment. Supplements of phosphate, electrolytes, vitamin B12, folate, vitamin D, magnesium and zinc are all readily available and of potential benefit with low risk. As there is the potential for neurological toxicity from manganese supplementation, dietary sources may be preferable. Whilst it is clear many of these abnormalities are caused by the EDs it is possible some could be pre-existing and contribute to the development of EDs. Further research is indicated to identify the pathways and abnormalities involved, and to investigate the role of specific nutritional supplementation in the treatment of EDs.

## Abbreviations

AN: Anorexia nervosa
AN/BN: Initial AN diagnosis then BN at a later stage
BN: Bulimia nervosa
EDNOS: Eating disorders not otherwise specified
EDs: Eating disorders
GP: General medical practitioner
RIs: Reference intervals
SD: Standard deviation
TG: Total study group patients
